# Confidence-Calibrated Human Activity Recognition

**DOI:** 10.3390/s21196566

**Published:** 2021-09-30

**Authors:** Debaditya Roy, Sarunas Girdzijauskas, Serghei Socolovschi

**Affiliations:** School of Electrical Engineering and Computer Science (EECS), KTH Royal Institute of Technology, 114 28 Stockholm, Sweden; sarunasg@kth.se (S.G.); serghei@kth.se (S.S.)

**Keywords:** wearable sensors, human activity recognition, deep learning, confidence calibration, time-series classification, signal processing, model reliability, training algorithm

## Abstract

Wearable sensors are widely used in activity recognition (AR) tasks with broad applicability in health and well-being, sports, geriatric care, etc. Deep learning (DL) has been at the forefront of progress in activity classification with wearable sensors. However, most state-of-the-art DL models used for AR are trained to discriminate different activity classes at high accuracy, not considering the confidence calibration of predictive output of those models. This results in probabilistic estimates that might not capture the true likelihood and is thus unreliable. In practice, it tends to produce overconfident estimates. In this paper, the problem is addressed by proposing *deep time ensembles*, a novel ensembling method capable of producing calibrated confidence estimates from neural network architectures. In particular, the method trains an ensemble of network models with temporal sequences extracted by varying the window size over the input time series and averaging the predictive output. The method is evaluated on four different benchmark HAR datasets and three different neural network architectures. Across all the datasets and architectures, our method shows an improvement in calibration by reducing the expected calibration error (ECE)by at least 40%, thereby providing superior likelihood estimates. In addition to providing reliable predictions our method also outperforms the state-of-the-art classification results in the *WISDM, UCI HAR, and PAMAP2* datasets and performs as good as the state-of-the-art in the *Skoda* dataset.

## 1. Introduction

Extracting data from wearable sensors and converting them into meaningful information has given rise to different paradigms in ubiquitous and pervasive computing. Human activity recognition (HAR) with sensors is one such paradigm that has gained much traction in the past 15 years. HAR is the method for classifying human activities in multiple contexts. Sports [1], personal fitness tracking [2], tracking activities of daily life (ADL) [3], and geriatric care [4] are some prominent applications of HAR with wearable sensors. Popular sensors used in this domain include accelerometer, gyroscope, magnetometer, heart-rate sensor, etc. HAR with wearable sensors is a multivariate time-series classification problem. In recent years, the most popular choice for modeling human activity recognition (HAR) problems has been deep learning (DL). The expressiveness of DL techniques towards automatic feature extraction made it a popular choice among researchers to investigate their applicability to the HAR problems. Most DL architectures strive to improve primarily on predictive accuracy, i.e., they focus on detecting the activity classes correctly during test time. However, in addition to providing a correct prediction, it is also essential to produce a reliable prediction. Calibration of confidence, i.e., predicting a probabilistic estimate representing the actual likelihood, plays a crucial role in this aspect. Calibration aims to reduce the gap between the predictive accuracy and confidence estimates of the prediction. Standard neural networks trained towards accuracy are prone to producing miscalibrated confidence estimates through the softmax function. In practice, they produce overconfident wrong estimates, thus affecting the reliability of the model [5]. Although calibrating neural networks has taken off in the last couple of years in computer vision and NLP domains [6,7] it is relatively under-explored in the context of HAR with wearable sensors. This paper bridges this gap by proposing a novel neural network ensembling method for wearable sensor-based HAR.

Model ensembling is a popular technique used in machine learning for improving classification metrics [8]. An ensemble reduces variance in predictions that improve classification accuracy. A reduction in predictive variance also results in a better calibration [9]. Thus, the right ensembling strategy improves both calibration and classification.

A discretized interval of time, also called a time window, is a crucial component of an HAR process. By extracting patterns from the temporal window, the DL model can classify one activity from another. In most previous work, fixed window size and a fixed overlap are used for datasets containing different activities exhibiting different patterns in the time series. For a homogeneous dataset containing a similar type of activity, the fixed window size pattern extraction is logical. However, the one size fits all concept is counter-intuitive for heterogeneous datasets containing different types of activities. The time window needed to extract features for one activity might differ from another activity (owing to nature, periodicity, etc.). e.g., the periodicity of a *running* activity is different from *vacuum cleaning*. To extract features using the same window size from these two very different activities is sub-optimal. To overcome this problem, an input-ensemble-based novel training method, *deep time ensembles* is proposed. In this method, different models trained with temporal sequences extracted using different window sizes from the raw signal are ensembled. The idea is that each model of the ensemble specializes in recognizing certain genre activities that are sensitive to the input sequence created with specific window size. An ensemble thus combines the individual expert predictors to boost the overall prediction capability. Moreover, the probabilistic estimates harnessed from each model vary due to the variable window sizes. Averaging them softens the softmax output of the ensembled models, thus eliminating the overconfident estimates produced by individual softmax functions. The combined effect of variance reduction, softening of softmax, and predictive boosting through ensembling helps *DTE* calibrate HAR models as well as improve its classification performance.

**Figure 1:**
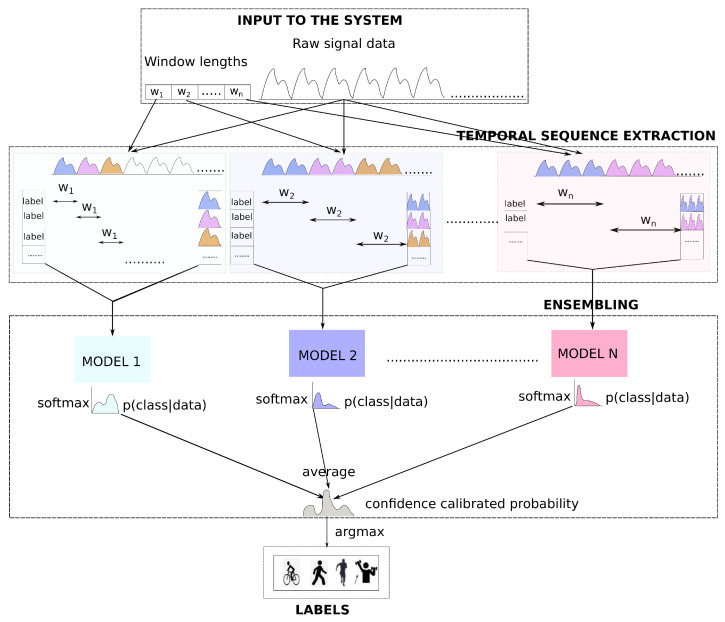
Deep time ensembles: Overview.

The proposed method has two main parts (see Figure 1): *temporal sequence extraction*, where based on a set of different window sizes, temporal matrices from the raw signal (with the window size and window overlap as hyperparameter) are extracted. In the second part *ensembling*, the temporal matrices are used to train individual models of the ensemble. The softmax output from each of those models is averaged to produce the final prediction.

*DTE* is evaluated on four public datasets, *WISDM* [10], *UCI* [11], *PAMAP2* [12], and *Skoda* [13]. *DTE* can be used in conjunction with any model architecture. In this paper, three architectures were chosen, *CNN*, *LSTM*, and *Convolutional LSTM*. *DTE* improves calibration by reducing the expected calibration error (ECE) by at least **40%** for all the datasets and architectures. Moreover, it is observed that *DTE* outperforms the baseline models on the classification metrics for all the chosen datasets (except Skoda) by at least **2%**.

The main contribution of the paper is the proposition of *deep time ensembles*, a simple yet effective ensembling technique for HAR with time-series data originating from wearable sensors. The system’s effectiveness in calibrating deep learning models and improving the classification metrics was demonstrated. For calibration experiments, the state-of-the-art models were replicated and compared with *DTE*. The method is also compared with the standard temperature scaling recipe [14] (used for improving calibration) and reflected upon the model performance. To the best of our knowledge, no previous work explored calibration in the context of HAR.

The remainder of the article is organized as follows: Section 2 discusses the proposed methods, where the training algorithm and the defined metric are discussed. Next, Section 3 presents an extensive evaluation. Then, Section 4 discusses related works, followed by a conclusion in Section 5.

## 2. Related Works

In this section, the literature is grouped into several categories, and a comparative analysis of the proposed method with respect to each group is presented.

### 2.1. Wearable Sensor-Based HAR Using Deep Learning

HAR from mobile and wearable sensor data have been studied using learning algorithms. Among the Machine learning methods, decision tree [15], random forest [16,17], and SVM [18,19] showed the best performance in the task; however, one of the main drawbacks of these approaches is the need for handcrafted features reliant on domain knowledge. It is often difficult to evaluate the extracted features’ efficiency since there is no defined strategy for their extraction. In addition, the procedures could be time consuming, and the extraction methods introduce assumptions about data, increasing the induction bias. In recent years, the most popular choice for modeling HAR problems has been deep learning (DL) [20,21,22,23,24]. The expressiveness of DL techniques towards automatic feature extraction made it a popular choice among researchers to investigate their applicability to the HAR problems. Naturally, a lot of robust architectures have evolved that have pushed the state-of-the-art in these problems. Due to their ability to capture temporal information, convolutional neural network (CNN) [25] and long short-term memory (LSTM) [26] based models have particularly proved to perform very well HAR with sensor data. This characteristic allows the neural networks to extract temporal features from the data using less preprocessing, thus reducing the learning bias and making the deep learning models more suitable for building end-to-end systems, facilitating both the training and recognition processes. Popular choices of deep-learning architectures for HAR encompass 1D convolutional neural networks [20], recurrent neural networks [27], including the LSTM variant, autoencoder-based architectures [28], and other hybrid solutions, such as convolutional LSTM [21]. While the deep-learning-based methods rely on a *fixed window size* to extract temporal sequences from time-series sensor data, *DTE* uses a number of different window sizes as input and trains a neural network ensemble. This helps boosting the classification metrics when compared to some previous works [6,20,29,30]. Furthermore, *DTE* can be used with any base neural network architecture. Table 1 presents the datasets, architecture, and macro f1-scores for some of the previous works that are chosen as baselines in this paper.

### 2.2. Calibration of Neural Networks

Guo et al. [14] argued that despite achieving very high accuracy the modern neural networks are poorly calibrated, affecting the reliability of the model predictions. This behavior could compromise the decision-making process in safety-critical systems. In the domain of computer vision, calibration has been explored recently [7,9,14]. To the best of our knowledge, confidence *calibration of predictive output has not been explored in HAR previously*. The devised method adapts calibrating DL models used in sensor-based HAR procedures. This improves the reliability of the models by generating predictions that represent the true likelihood.

Many approaches were proposed to adjust the model calibration, from tuning the model capacity, weight regularization, and batch normalization to stand-alone methods that require a hold-out validation set for hypertuning. Among the latter, two main families of algorithms can be identified, inspired by histogram binning or Platt scaling [31] algorithms. The first family includes histogram binning [32], isotonic regression [33], and Bayesian binning into quantiles (BBQ) [34]. The second one contains matrix scaling and temperature scaling [14]. All these methods require an extra post-processing step. *DTE* can be used to calibrate predictions from time-series (in this paper for HAR)-based neural network models.

### 2.3. Ensembling

Inspired by ensembles used in uncertainty estimation [35], *DTE* is formulated that achieve well-calibrated confidence estimates without any extra post-processing. While ensembles have been used previously in the context of classification [36,37], the research closest to our work can be found in Guan et al. [6]. They propose an ensemble of LSTM learners to achieve HAR; however, the window-size selection for temporal sequence extraction is different compared to our method. Additionally, our primary goal is to explore model calibration, which was not taken into consideration by [6].

## 3. Methods

In this paper a supervised time-series classification problem is addressed. Our objective of calibration is similar to that of [14]. The input data are *x*, and the corresponding labels are *y*. A neural network *f*, parameterize the distribution $p_{\theta }\left(y|x\right)$. The confidence of the predictions is given as *P*. A calibrated output $\hat{P}$ is desired, such that it represents the true probability. By producing a calibrated output, the gap between the accuracy and mean confidence of the model is also reduced. For a perfectly calibrated model, for mean confidence of 0.8, a mean accuracy of 0.8 is expected.

To visualize calibration *reliability diagram* is used [38]. The reliability diagram representation is adopted from [39]. In these diagrams accuracy is plotted as a function of confidence (see Figure 5a). In the upper part of Figure 5a the confidence is represented in the *x*-axis and accuracy is represented in *y*-axis. Furthermore, the *x*-axis is divided into fixed number of intervals (10 in this case). Predictions that fall in the confidence interval are assigned to that bin. After assigning the predictions, the mean confidence and the mean accuracy of the individual bins are calculated. For a multi-class classification problem, the accuracy of each bin is given by,

(1) $$\begin{array}{c} acc\left(B_{m}\right)=\frac{1}{n}\sum_{i\subset B_{m}}argmax\left(p_{\theta }\left(y_{i}|x_{i}\right)\right) \end{array}$$

and the average confidence of each bin is defined as,

(2) $$\begin{array}{c} conf\left(B_{m}\right)=\frac{1}{n}\sum_{i\subset B_{m}}max\left(p_{\theta }\left(y_{i}|x_{i}\right)\right) \end{array}$$

where *n* is number of samples in each bin, and $B_{m}$ is the bin number. The bold black line represents the accuracy of each bin in the *confidence versus accuracy plot* (top part of the diagram). The bars represent the difference between the accuracy and the confidence in each of the bins. The lower part of the reliability diagram displays the histogram of the samples assigned to each bin and helps understand the importance of calibration in each of the bins. e.g., in Figure 5a most of the samples are concentrated in the last bin, and the calibration of that bin has more impact on the overall calibration. These two boxes, when seen in conjunction, provide us with a holistic understanding of model calibration. An identity function is plotted for a perfectly calibrated model (denoted by the diagonal line on the upper part). The miscalibration is represented by deviation from the perfect diagonal.

While reliability diagrams are a visual explanation towards calibration, expected calibration error (ECE) is a metric-based representation of the same. Motivated by the fact that miscalibration is the difference between confidence and accuracy, ECE captures the weighted average difference of bin’s accuracy and the confidence score. It is given as:

(3) $$\begin{array}{c} ECE=\sum_{m=1}^{M}\frac{n_{m}}{N}\left|acc\left(B_{m}\right)-conf\left(B_{m}\right)\right| \end{array}$$

where *M* is the number of bins and *N* is the number of samples.

### 3.1. Ensembles and Model Calibration

There is an intrinsic connection between predictive variance, accuracy, and calibration. In their work, Seo et al. [9] showed that predictive variance is inversely proportional to both calibration and accuracy. A good ensembling regime helps to reduce the variance in prediction and boost the predictive performance [40]. Thus, it is hypothesized that reducing predictive variance through ensembling helps model calibration and classification. In comparison with *temperature scaling* [14], a popular calibration method, ensembling does not require any extra post-processing round.

Furthermore, traditional neural networks trained towards softmax are prone to be miscalibrated. This is primarily attributed to the overestimated probability assignment of the positive class by the softmax function [5]. Using these neural network models can result in overconfident probability estimates at the output. Ensembling, equivalent to Bayesian model averaging, helps to incorporate uncertainty in the data effectively [35]. In ensembling, the softmax output from individual models is softened through averaging, mitigating the overconfident outputs of individual models. Hence, a well-calibrated probability distribution is obtained at the output. This is reflected in the predictions as well. The observations about ensembling led us to devise a novel ensembling method fit for time-series-based HAR called *deep time ensembles (DTE)*.

### 3.2. Deep Time Ensembles

**Figure 2:**
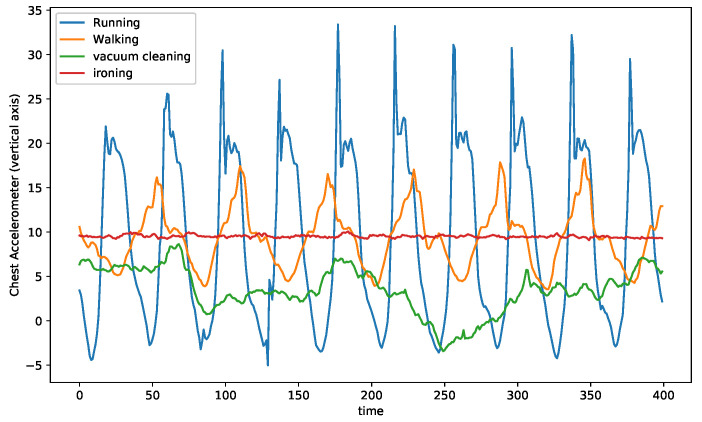
Comparison among three activities from PAMAP2 dataset.

Time series recordings have the structural information encoded into their temporal order. The data fed into the model are strictly ordered by the acquisition time. The scope of exploration depends explicitly on the number of consecutive values fed into the model. Hence, extracting the temporal information is highly dependent on the window size of sensor readings and the overlap between the consecutive windows. These two hyperparameters influence the order of dependencies between empirical values explored by the model. Traditionally in activity recognition, temporal sequences are extracted from raw time-series signals using a fixed window size. These temporal sequences and their corresponding labels for each sequence are used for training the deep learning models. For datasets that consist of homogeneous activities, i.e., activities that exhibit similar patterns in their signal representation, a fixed window size might suffice; however, in datasets with a wide range of dissimilar activities, the fixed window size is sub-optimal. In Figure 2, the chest accelerometer data of four activities (out of twelve) from the PAMAP2 dataset for a single subject are plotted.

On the one hand, the *running* and *walking* activities have a visible periodicity. On the other hand, the *vaccum cleaning* activity has much less evident periodicity, while the *ironing* activity has almost no periodicity. While for the *running* and *walking* assuming a window size equal to the period or two periods of the activity for temporal sequence extraction, it might be the right choice for the DL model; however, the same window size is rather long for pattern extraction of a static activity such as *ironing*. Similarly, *vaccum cleaning* requires a different optimal window size. Thus, each activity in the same dataset is sensitive to a different choice of window size that benefits the learner.

Based on the above observation and our goal of calibrating HAR models with an ensemble, the *deep time ensembles* method is proposed. In our algorithm, temporal sequences from the same input signal using different window sizes is extracted. For each window size a collection of temporal sequences form the temporal matrix. Thus multiple temporal matrices are extracted with multiple window sizes. With the extracted temporal matrices, an ensemble of models is trained (one model per temporal matrix). Each model in the ensemble would be expert at recognizing certain activities, and the combination of all would be beneficial for the overall HAR model. Creating an ensemble with fixed window-size duration would provide a limited amount of temporal information conveyed to the model, and *DTE* is superior in that aspect. Furthermore, the extraction of temporal sequences with different windows size allows us to model the uncertainty beyond the length of window size. Eventually, by averaging the predictive response over the ensemble, it is possible to model the uncertainty that depends solely on the recordings and no other hyperparameter (i.e., window size). The promotion of such coherent (recording) uncertainty helps mitigate the overconfidence that might come from the softmax distribution of a single model. This, in turn, helps to calibrate the likelihood coming out of the ensemble.

*DTE* has two main steps

- Extracting *temporal sequences* from the time series (See *temporal sequence extraction* module of Figure 1) based on a set of different window sizes.
- Training ensembles based on extracted temporal sequences (See *ensembling* module of Figure 1).

The goal of the machine learning method is to determine the activity given a sequence of the wearable sensor signal. As seen in Figure 1, there are two inputs to our system, the *raw signal data*, originating from sensors, and an *array of window sizes* to extract temporal sequences for individual models of the ensemble. The overlap between windows is another input to our model, but it is considered as a fixed value for the method and is omitted in the diagram. The same data serve as input for each block inside the *temporal sequence extraction* module. In the first block, a sliding window of size $w_{1}$ is selected and slided over the raw signal data continuously until the data expire. For each slide of $w_{1}$, extract temporal sequences from the raw time-series data are extracted. All those temporal sequences (until the end of the data) are appended to form a temporal matrix. There is a related activity for each temporal sequence, and the extracted labels for all temporal sequences form the label vector. Similarly, a window size of $w_{2}$ is used for the next block and this creates another set of temporal matrices and label vectors. In this way, temporal matrices up to $w_{n}$ are extracted. For each of such temporal matrices and the corresponding label vector, a neural network model (with the architecture of our choice) is trained in the *ensembling* module. During the prediction/evaluation, the softmax distribution output from each model of the ensemble is averaged. The averaged distribution is a confidence-calibrated one, and the index of the maximum value of the distribution gives us the activity label.

Algorithmically, *DTE* is divided in two phases, the *training* phase and the *evaluation/prediction* phase. The *training* phase happens following the *temporal sequence extraction*. Once individual temporal matrices and label vectors are extracted for each selected window size, the training is similar to any other DL method. The algorithm is presented in Algorithm 1.
**Algorithm 1** Deep Time Ensemble—Training1:A neural network parameterize a distribution $p_{\theta }\left(y|x\right)$. It is trained with cross entropy loss.2:One dimension of a signal is represented by *n* sensor recordings $s_{1},s_{2},\ldots ,s_{n}$.3:There are *n* labels $l_{1},l_{2},\ldots ,l_{n}$ where $l_{j}$ is the label for sensor recording $s_{j}$4:Select *N* window sizes representing different time duration $w_{1},w_{2},\ldots ,w_{N}$5:Select a overlap6:**for**i=1 to *N* **do**7:    **for** j=1 to *n* **do**8:        start=j9:        $end=start+w_{i}$10:        Append temporal sequence from start to end to temporal matrix $X_{i}$.11:        Append label temporal sequence (e.g., most occurring activity) label vector $L_{i}$12:        j+= overlap until the signal ends.13:    **end for**14:    Train $p_{\theta_{w_{i}}}\left(y|x\right)$ with $X_{i}$ and $L_{i}$.15:**end for**

**Figure 3:**
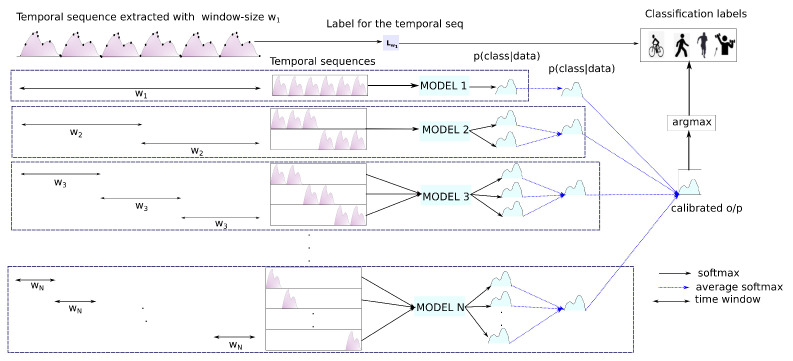
Magnified view of DTE evaluation for one temporal sequence.

There are specific considerations that are required while performing an evaluation/prediction with *DTE* (depicted in Figure 3). In the diagram, a single temporal sequence extracted with window size $w_{1}$ is presented. For the sake of convenience, it is called $t_{1}$. The temporal sequence consists of $w_{1}$ recorded sensor points. There are *N* models pre-trained with temporal matrices and labels extracted with $w_{1},w_{2},\ldots ,w_{N}$ windows, where $w_{1}>w_{2}>\ldots >w_{N}$. While training and evaluating, each temporal sequence of all the temporal matrices is associated with a single label. In the demonstrated figure, the label index for the temporal sequence is given as $L_{w_{1}}$. For the window size $w_{1}$, the whole temporal sequence is fed to the model (MODEL 1 in the figure) trained on a temporal matrix created with $w_{1}$. Using window size $w_{2}$, two temporal sequences from $t_{1}$ can be extracted. Both these temporal sequences are used to generate two softmax distributions from MODEL 2 that are averaged out as a single distribution. In the diagram, overlapping windows are omitted for the sake of simplicity.

Similarly for $w_{3}$, three temporal sequences are extracted from $t_{1}$, and an averaged softmax is extracted from the MODEL 3. The process is continued all the way up to $w_{N}$, the smallest window size. Finally, the single distributions that are obtained earlier are averaged to output the confidence calibrated probabilities. During the evaluation, the label corresponding to the index of the maximum value of the distribution is evaluated with the actual label $L_{w_{1}}$. While in a live-system the confidence calibrated output can be provided as the result. The Algorithm 2 extends the same concept to multiple temporal sequences or a temporal matrix $X_{1}$ extracted with window size $w_{1}$.
**Algorithm 2** Deep Time Ensemble—Evaluation/Prediction1:**Inputs:***N* window sizes, temporal matrix $X_{1}$ of size $m*w_{1}$, label vector $L_{1}$, *N* neural network models represented by $p_{\theta_{w_{i}}}\left(y|x\right)$.2:**for**i=1 to *m* **do**3:    **for** j=1 to *N* **do**4:        **if** j=1 **then**5:           Predict softmax distribution $y_{ij}$ with model $p_{\theta_{w_{j}}}\left(y|x\right)$ with $X_{1_{i}}$.6:        **else**7:           Extract temporal sequence matrix $X_{j}$ by sliding $w_{j}$ on $X_{1_{i}}$8:           **for** Every row in $X_{j}$ **do**9:               Predict softmax distribution $y_{j,row}$ with model $p_{\theta_{j}}\left(y|x\right)$ with row as input.10:           **end for**11:           $y_{ij}=\frac{1}{nrows}\sum_{k=1}^{nrows}y_{j,row}$12:        **end if**13:    **end for**14:    Combine predictions from all the ensembles as $p_{i}\left(y|x\right)=N^{-1}\sum_{j=1}^{N}y_{ij}$15:    If evaluating match argmax ($p_{i}\left(y|x\right)$) with label at index $L_{1_{i}}$.16:**end for**

The novelty of this paper is two-fold:

1. Dissimilar activities are associated with different time windows instead of a fixed one as proposed in most of the earlier works. This led to devising *DTE* that ensembles different temporal representation of the same input signal.
2. The observation that the ensembled model also calibrates the predictive output. This in turn results in predictions that represents the true likelihood.

In the next section an extensive evaluation of *DTE* is presented that justifies the mentioned formulations.

## 4. Evaluation

To validate the effectivness of our method in calibrating HAR models, a range of experiments were conducted on four datasets, namely *PAMAP2*, *UCI*, *WISDM*, and *Skoda*. For the experiments, three neural network architectures were chosen, namely *CNN*, *LSTM*, and *convolutional LSTM* (discussed in methods). The architectures for each were chosen so that they match the previous works that are compared with this paper, and they are presented in Table 1. The previous works and the corresponding architectures are replicated to form a baseline for comparison with *DTE*. Primarily, *DTE* is evaluated on three factors.

1. How does *DTE* fare in calibrating neural network models?
2. How does *DTE* compare with the popular temperature-scaling [14] method of calibration?
3. How does *DTE* compare with the previous work in the downstream classification task?

To evaluate the calibration, the standard metric is expected calibration error (ECE), as defined in the methods section. In particular, we strive for a lower value of **ECE**, since it means that the predictions are more calibrated or representative of the true probability. The metrics used for evaluating the classification performance of our method are **accuracy**, **macro f1 score**, and **average f1 score**. In rest of the section: the datasets are described first, followed by model configuration, then the calibration results are discussed followed by classification performance.

### 4.1. Datasets

**Figure 4:**
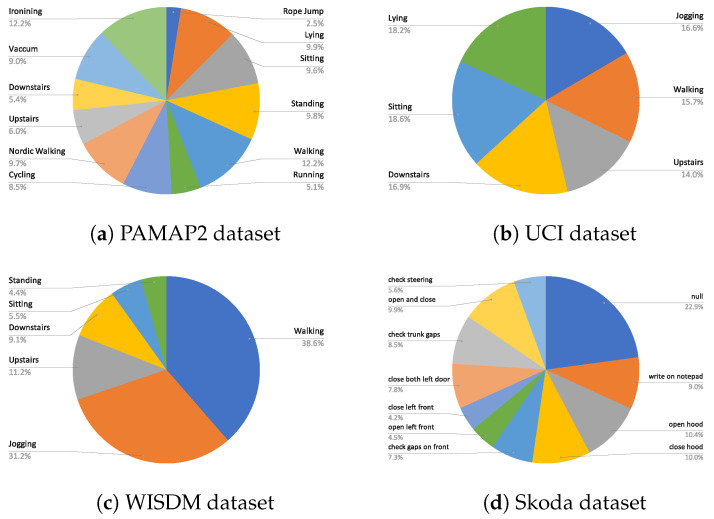
Class distribution of (**a**) PAMAP2, (**b**) UCI, (**c**) WISDM, and (**d**) Skoda dataset.

The chosen datasets consist of a good mix of different activities and sensor modalities. The class distribution of the activities in each dataset is shown in Figure 4. Next, the specifics of each of the datasets are highlighted.

- **WISDM dataset:***WISDM* dataset [10] consists of 36 subjects and 6 activities, namely *standing, sitting, downstairs, upstairs, walking, and jogging*. The activities were recorded with a tri-axial accelerometer sensor. The training, validation, and evaluation splits for the *WISDM* dataset are adopted from [20,29]. Users 1–24 form training data, 24 and 25 form the validation data, and 26–36 are used for testing. After preprocessing and windowing of the test split, 3026 test samples for evaluation are obtained.
- **UCI dataset:** The *UCI* dataset [11] is a public dataset consisting of six activities *lying, standing, sitting, downstairs, upstairs, and walking* recorded from 30 subjects. The dataset was recorded with a triaxial accelerometer and gyroscope, resulting in six dimensions. Similar to the *WISDM* dataset, the training, validation, and testing split of this dataset was also adopted from [20]. After preprocessing and windowing, the number of test samples for evaluation is 2993.
- **PAMAP2 dataset:** The *PAMAP2* dataset [12] consists of 12 activities recorded from nine subjects for over 10 h. It consists of sporting activities, activities of daily life, and other domestic activities. It consists of a wide array of multivariate sensor data (accelerometer, gyroscope, magnetometer, heart rate, etc.), resulting in 52 dimensions. The training, testing, and validation dataset was extracted following the protocol of [30]. Runs 1 and 2 from subject 5 is the validation set, and runs 1 and 2 from subject 6 is the testing set. The rest of the data were used for training. Guan et al. [6] did a thorough sample-wise evaluation on the *PAMAP2* dataset in their work. To accommodate a similar evaluation strategy, testing samples from the testing dataset with complete overlap are extracted. This gives 83 K samples for testing. These samples are used to evaluate our method with both [6,30].
- **Skoda dataset:** The *Skoda* dataset [13] is comprised of a collection of 10 manipulative gestures/activities of a factory worker working in the assembly line of a car manufacturing process. The worker wore 20 3D accelerometer sensors. The training/validation/testing splits of the *Skoda* dataset are adopted from [21]. To create test samples same overlap as training is assumed.

For *DTE*, there are multiple window sizes that are used to extract temporal sequences from the same input signal. This leads to different sizes of training and label samples for each model of the ensemble. In Table 2, the number of temporal sequences that are extracted from the same dataset, for different window sizes is presented.

### 4.2. Model Configuration

In this work, a baseline neural network model was chosen, *DTE* was applied to it, and the baseline was compared with its *DTE* variant. The neural network model configurations were chosen from previous works and became baselines for making a fair comparison. For *PAMAP2* dataset, the *CNN* configurations from [30], and the *LSTM* configurations from [6] were adopted. For the *UCI* dataset, the model parameters of *CNN* were chosen from [20]. Although [20] added an extra feature layer as concatenation in their work, it is omitted in our baseline. This is to keep the feature extraction procedure as automated as possible. For the *LSTM* architecture of the *UCI* dataset, a two-layer *LSTM* with 128 neurons in each cell is selected. The *LSTM* configuration of the *WISDM* dataset was chosen from [29], and for the *Skoda* dataset, the convolutional *LSTM* architecture has the same parameters as [21]. Table 1 lists the baseline models that were considered for applying *DTE*. Throughout, the evaluation the results of calibration and classification on these architectures are presented. The details of the architectures and hyperparameters are presented in Appendix A.

### 4.3. Calibration Results

To the best of our knowledge, no previous experiments on the calibration of neural networks on HAR were showcased. Hence, the previous works stated in Table 1 are replicated and the **ECE** metric is calculated by adding the calibration module to the replication. The previous works are called *baseline models* for the rest of the section.

#### 4.3.1. ECE and Reliability Diagrams

**Figure 5:**
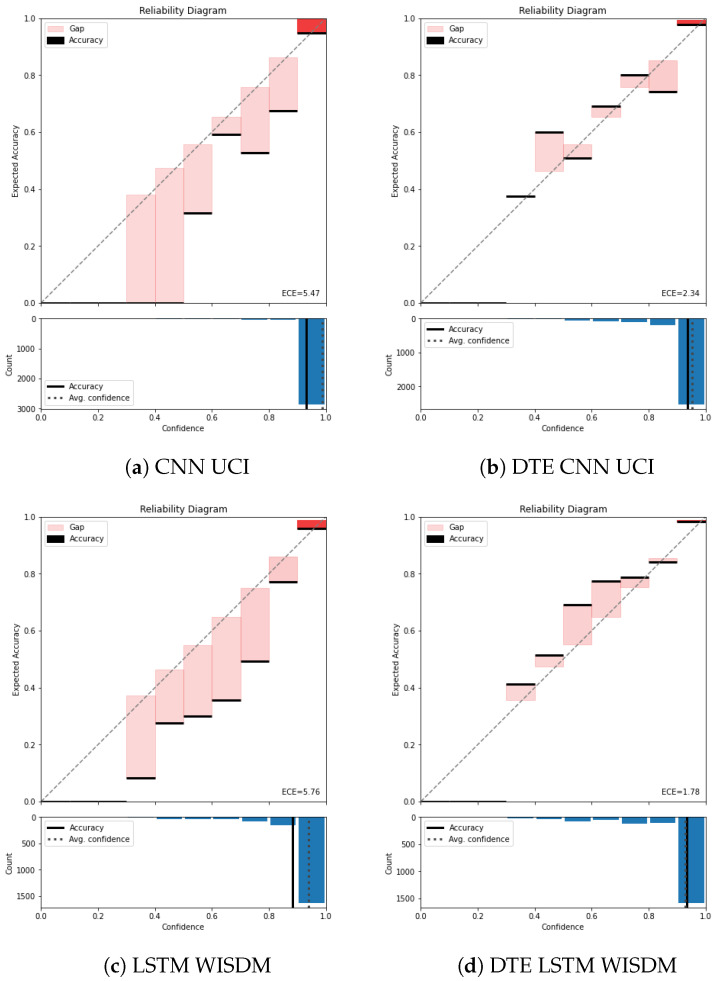
*Reliability diagram**UCI**and**WISDM**: Without temperature scaling*. The top half of each reliability diagram (**a**–**d**) has *confidence* in the *x*-axis and *accuracy* in the *y*-axis. The *x*-axis *(confidence)* is divided into 10 bins. The colored bars represent the difference between mean accuracy and the mean confidence of the samples that falls in those bins. The black line on top or bottom of each bar represents the accuracy of the samples in that bin. The bottom half of the reliability diagram represents the histogram of the samples concentrated in each bin. The **ECE** is calculated be averaging the difference between confidence and accuracy across all the bins.

As discussed earlier, the standard measure of calibration is ECE. An essential factor for calculating ECE is selecting the number of bins over which the metric will be calculated. The number of bins were chosen to be 10 (M=10) across all the experiments and architectures for our experiments. The calibration result is reported in Table 3. In this table, the ECE for all the baseline architectures and the corresponding *DTE* variants for each dataset are presented. All experiments were run 10 times for robustness and the mean and the standard deviation on the metrics are shown. From Table 3 it is observed that the baseline model for *PAMAP2* dataset adopted from Hammerla et al. [30] has an ECE of 0.06, while applying *DTE* on it decreases the ECE to 0.03. This drop in ECE indicates an improved calibration. Similarly, across all the dataset and architectures, *DTE* improves calibration by at least 40%. The visual aid for understanding calibration is a reliability diagram. The reliability diagrams for the baselines and *DTE* for all the datasets are plotted. e.g., in Figure 5 the reliability diagram for the *UCI* dataset for the chosen architectures (*CNN* and *LSTM*) is presented.

Color temperature is used to denote the gap bars (difference between confidence and accuracy) based on the number of samples residing in each bin. In Figure 5a it is observed that the baseline *CNN* model is highly confident about most of the predictions and has put most of the examples in the highest bin (between 0.9 and 1).

The average confidence of this model is almost 1.0 (seen by the dotted line in the lower part of the reliability diagram), while the accuracy is approximately 0.93. This gap between the average confidence and the average accuracy represents the miscalibration of the model. The average binned miscalibration is captured through **ECE** value of 5.47. Meanwhile in Figure 5a, on applying *DTE* with the same architecture results in a substantial drop in **ECE** to 2.34. It is also noted that the gap between accuracy and the confidence in the lower part of the reliability diagram has reduced. Zooming into the highest bin for both the baseline model and the *DTE* variant highlights that the gap between accuracy and the confidence is lower in the *DTE* variant compared to the baseline *CNN*. This is also observed for *LSTM* variants of *UCI* dataset (Figure A2a,b)

**Figure A1:**
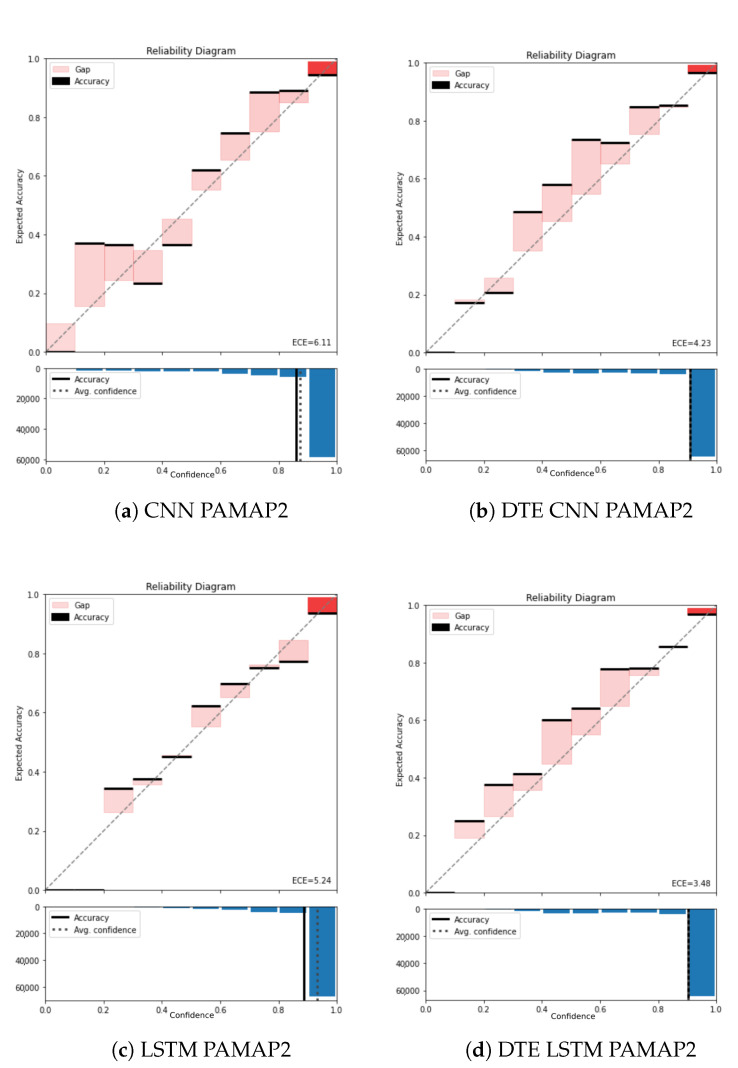
Reliability diagram of *PAMAP2* for (**a**) CNN, (**b**) DTE CNN, (**c**) LSTM, and (**d**) DTE LSTM.

**Figure A2:**
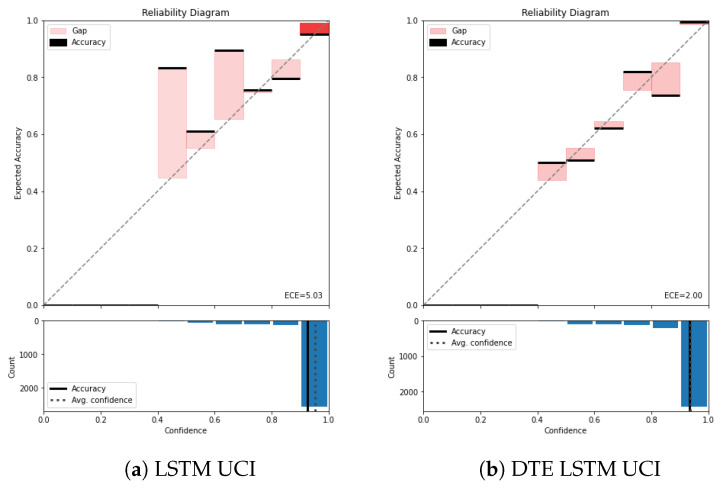
Reliability diagram of *UCI* for (**a**) LSTM and (**b**) DTE LSTM.

**Figure A3:**
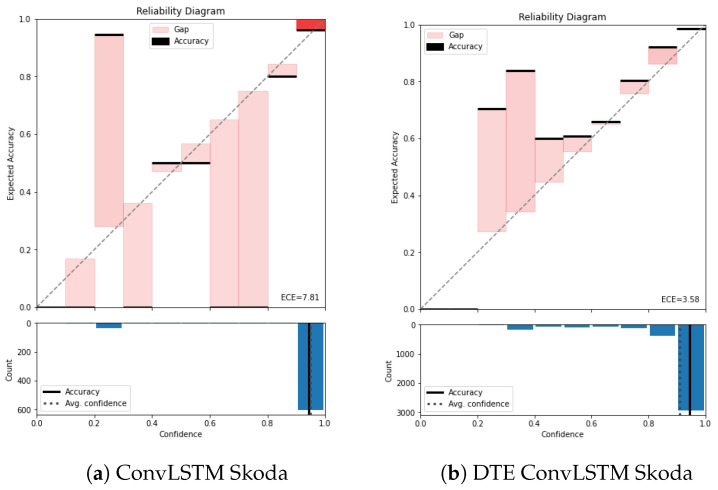
Reliability diagram of *Skoda* for (**a**) ConvLSTM and (**b**) DTE ConvLSTM.

Another observation is that *DTE* distributes test examples from the highest bin to the lower bins through the averaging procedure. This helps in bringing down the overall average confidence of the model. This is in line with the hypothesis, *DTE* mitigates the overconfidence achieved by softmax through uncertainty estimation (Bayesian model averaging) and variance reduction. The best value of calibration error is observed for *DTE LSTM* architecture in *WISDM* dataset. This can be attributed to the relatively simpler neural network architecture and lower-dimensional features of the dataset (model complexity is directly proportional to calibration error [14]). The reliability diagram for rest of the datasets are in Appendix B. Across all the datasets and architectures, *DTE* appears to be more calibrated than the baseline models (see Table 3 for overall results and Figure A1 for *PAMAP2*, and Figure A3 for *Skoda*).

#### 4.3.2. Binwise Calibration

**Figure 6:**
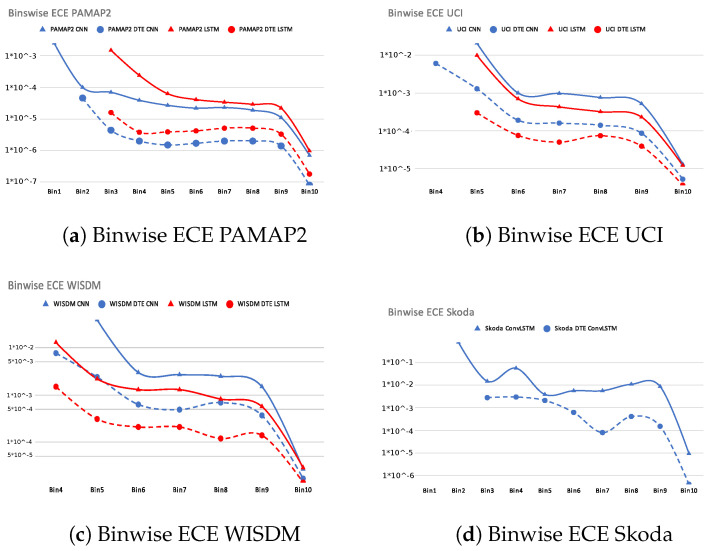
Binwise ECE (along *y*-axis) of all datasets and selected architectures.

Our calibration experiments showed that *DTE* decreases **ECE** for all the cases and is very well calibrated in the higher bins, where most of the examples are concentrated (depicted by the almost non-existent gap in the higher bins of the reliability diagrams of *DTE*); however, a good calibration regime must guarantee that all the bins are better calibrated than baseline models. Hence, it is imperative to present the results comparing the binwise calibration of the baseline models and *DTE* for the best architectures across all the datasets in Figure 6. The *x*-axis of Figure 6 is the bin number, and the *y*-axis is the log of **ECE** in each of the bin. The dotted lines show the results for all the variants of *DTE*, while the solid line of the same color depicts the result for the baseline model. Taking *PAMAP2* dataset as an example, the solid blue line represents binwise **ECE** of the baseline *CNN* model and the corresponding *DTE* variant is shown in the dotted blue line. It is seen that *DTE* exhibits lower **ECE** in all the bins than the baseline. This is true for all the datasets and architectures in Figure 6. This experiment verifies that not only in the bins where most of the examples are concentrated, but *DTE* also exhibits a lower calibration error in all the bins.

#### 4.3.3. Comparison with Temperature Scaling

**Figure 7:**
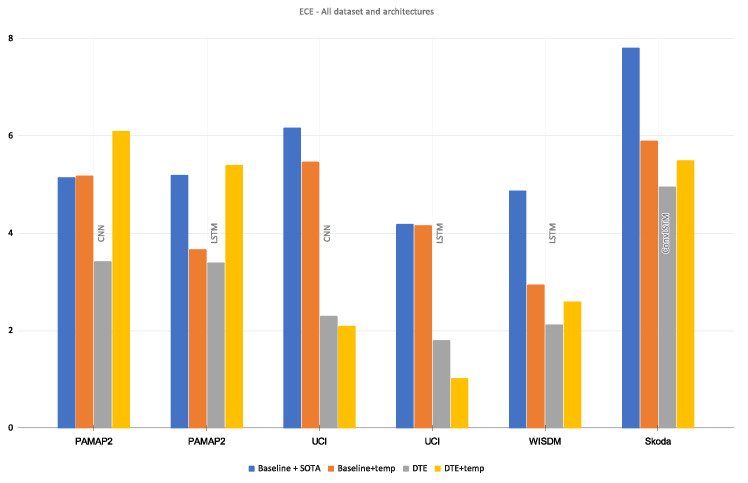
Comparison of **ECE** (along *y*-axis) between baseline, baseline + temperature-scaled, DTE, DTE + temperature-scaled models.

Apart from the comparison between *DTE* and the baseline model, the proposed method is also compared with *temperature scaling*. Specifically it is a comparison among baseline, baseline with temperature scaling, *DTE*, and *DTE* with temperature scaling. Temperature scaling is a post-processing method that aims to optimize a *temperature, T* based on a validation set. This *T* divides the softmax output and mitigates the overconfidence resulting in better calibration. To soften the softmax output, *T* must be greater than 1. The initialization of the *T* and the selection of the validation set is crucial for the optimization. If the validation set does not include all the classes, then the temperature scaling might give a sub-optimal solution (*T* < 1). The process is also very sensitive to the initialization of *T*. Following the initialization scheme found in the implementational details of the paper [14], the chosen temperature is T=1.5. With this temperature value and an optimal validation set, the softmax output of the baseline model and *DTE* is smoothed for our best architectures for all the datasets. The results are presented in Figure 7. With temperature scaling the baseline models were successfully calibrated; however, the scores indicate using only *DTE* results in better calibration than baseline with temperature scaling. With temperature scaling on the output of *DTE*, calibration only improved for the *UCI* dataset. For the rest of the datasets and architectures, *DTE* proved to be the optimal choice for achieving the best calibration.

This means that for most of the *DTE* models, suboptimal temperature values were reached through the optimization. This leads to a logical question about whether a different temperature initialization was required per model in the ensemble. This question is out-of-scope for this work; it can be explored in the subsequent extensions of this method. While temperature scaling is a popular method, the experiments show that *DTE* alone can calibrate effectively. Moreover even temperature scaling is used, it performs best when combined with *DTE* The reliability diagrams of temperature scaled variants are presented in Appendix B.

### 4.4. Classification Results

**Figure 8:**
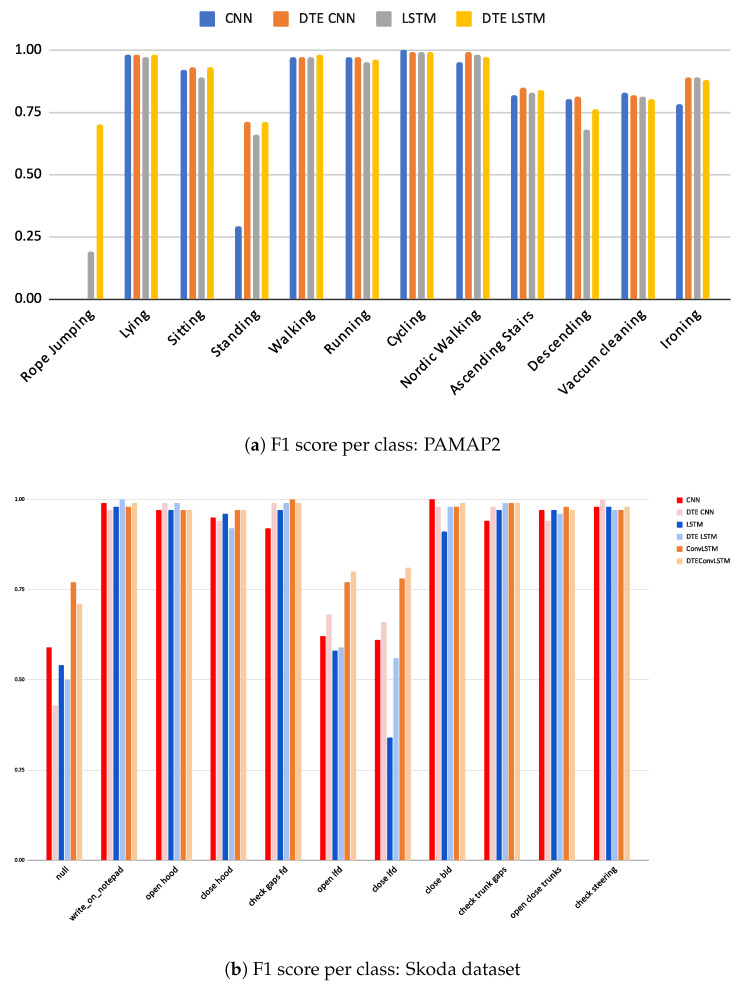
F1 scores per class for PAMAP2 and Skoda dataset.

Since HAR processes are primarily concerned with classification, it is essential to justify calibration while keeping the classification performance as good as possible. The classification results of the adopted baselines and the corresponding *DTEs* are presented in Table 3. A comparison between the baseline model and its *DTE* variant per dataset (e.g., *CNN* versus *DTE CNN*, *LSTM* versus *DTE LSTM*) is made and the best metrics for the whole dataset are highlighted. Furthermore, the class-wise F1-score for two datasets (rest are presented in Appendix B) are in in Figure 8.

**Figure 9:**
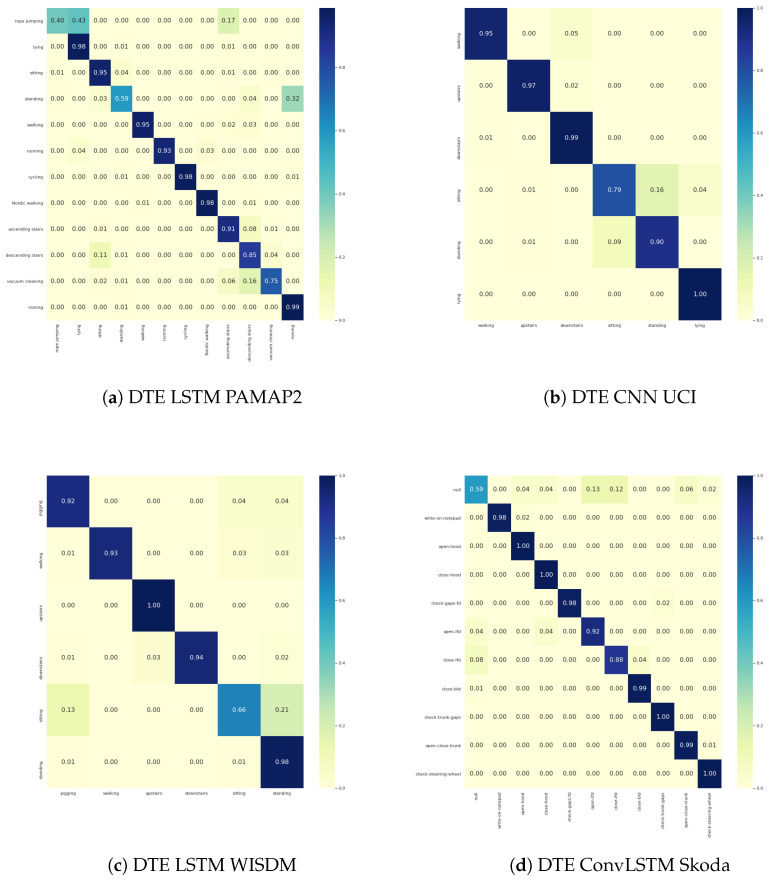
Confusion matrices for best *DTE* architectures.

The confusion matrices of the best-performing *DTE* variants per dataset are presented in Figure 9. For the sake of brevity, the rest of the confusion matrices are in the Appendix section. Analyzing the classification results exposes several exciting facts about *DTE*. From Figure 3 it is evident that every *DTE* variant consistently outperforms the baseline variants in all classification metrics across all the datasets (except F1${}_{w}$ in *ConvLSTM* architecture of *Skoda*).

This consolidates our argument that incorporating multiple models trained with different temporal matrices as an ensemble improves the classification performance. Unlike [6], the window sizes are selected in a non-random fashion. Although this has a manual constraint, our method ensures that each base model is a strong learner for a certain set of activities and performs adequately for the rest.

**Figure 10:**
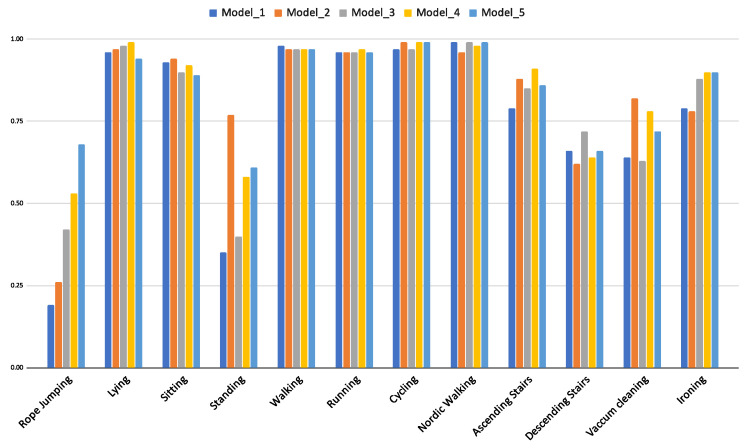
F1-score of activities per model that are trained on different window sizes in DTE using LSTM architecture for PAMAP2 dataset.

The idea of individual expert models contributing to the overall classification performance is demonstrated with an experiment where five individual models of *DTE LSTM* trained on *PAMAP2* dataset are compared. Figure 10 demonstrates how each of the five models that are created with ascending window sizes (5 s to 9 s) perform in detecting individual activities. Thus, *Model_1* is trained with temporal sequences obtained using window-size of 5 s, *Model_2* with temporal sequences obtained using window-size of 6 s and so on, up to *Model_5* that is trained on sequences acquired using window-size of 9 s.

The *rope-jumping* activity is best detected by *Model_5*, but it is outperformed in detection of the *standing* activity by *Model_2*. Thus, it can be inferred that no single model is an expert in detecting all the activities, rather a combined predictor is the optimal choice to detect all activities (evident from overall classification results in Table 3).

In the *PAMAP2* dataset it is observed that accuracy and F1${}_{w}$ for *CNNs* and *LSTMs* are almost equal and the F1${}_{m}$ is substantially better in the *LSTM* variants. It is important to note that the *rope-jumping* class in the *PAMAP2* dataset is underrepresented (2.5% of the training data and 0.1% in testing data). This class is not captured by *CNN* or its *DTE* variant but by both the *LSTM* variants (in Figure 8a). Since F1${}_{w}$ makes a weighted average calculation, this class being significantly less in number is obscured by the better predictions of the other classes; however, F1${}_{m}$ is a non-weighted average of the F1-score, and the inability of a model to capture one class is amplified with this score. Thus, the *CNN* models that cannot capture this exhibit a lower F1${}_{m}$ compared to the *LSTM* models. Hence, for this dataset, F1${}_{m}$ captures the accurate picture, and *DTE LSTM* outperforms all other baselines by at least 0.4.

For *UCI* dataset performances of *DTE* variants of *CNN* and *LSTM* architectures are similar, with *DTE CNN* outperforming the *DTE LSTM* by 0.1 in accuracy. The classwise f1 scores (see Appendix C) show that *DTE CNN* detects the running and downstairs activity better than the other variants. In this dataset, *DTE* improves the baselines by at least 0.1. Considering the baseline metrics were already high (>0.91), this improvement is substantial. Furthermore, as stated in the earlier section, the predictions are more calibrated in the *DTE* variants.

*WISDM* dataset exhibits similar classification trends as the *UCI* dataset; however, the performance gains are more significant in this case. The class-specific scores show that *DTE* variants outperform the baseline models in all classes except *sitting* where the *LSTM* baseline has slightly better score than *DTE LSTM*.

Among the three baseline architectures for *Skoda* dataset, the *ConvLSTM* architecture and its *DTE* outperform all others by at least 0.6 in all classification measures; however, unlike other datasets and architectures the *DTE* variant (*DTE ConvLSTM*) does not improve all the metrics of the baseline. The substantial presence of null class and its detection by *ConvLSTM* architecture result in a better **F1**${}_{w}$ (see Figure 8b) than *DTE ConvLSTM*. *DTE* is based on the idea that if the dataset consists of a wide range of different activities, each model would have the expertise to capture a specific genre of those activities. The ensemble, when combined, becomes an expert at recognizing all. A close observation reveals that the activities of the *Skoda* dataset in Figure 4 are somewhat similar; thus, the hypothesis that each model extracts specific class patterns through different temporal sequences is not very strong. This might be another fundamental reason that *DTE* exhibits lower score in **F1**${}_{w}$ for the *ConvLSTM* architecture in Skoda. Among the rest of the architectures in *Skoda*, *DTE* variants outperform the baseline variants.

Overall *DTE* improves the baselines in classification performance and produces well-calibrated predictions that are much more representative of the true probability. It also validates the hypothesis that datasets with a wide range of activities (*PAMAP2*) benefit the most from the incorporation of *DTE*. Our range of experiments also provides a soft guideline for architecture selection for different datasets—e.g., in *PAMAP2* and *WISDM*, *DTE LSTM* has best classification results, while for *UCI*, *DTE CNN* outperforms the rest and for *Skoda*, both *ConvLSTM* and its *DTE* variant performs equivalently. In cases where the baseline models are not beaten by *DTE*, the performance is almost similar but with better calibration. This resonates with our initial promise of providing good classification performance with well calibrated predictions.

### 4.5. Comparison with Standard Ensemble Models

While comparison with previous works demonstrated the effectivity of *DTE*, most of the compared methods (except [6]) were non-ensemble methods. Hence, for a more fair comparison *DTE* is compared with a neural network ensemble created with standard architectures (LSTM, ConvNets, etc.). The ensembling strategy adopted is similar to the ones in [35]. The neural network architecture is ensembled in the same parameter space, and the number of models is kept the same as the number of input window sizes in *DTE*. The results of the experiment are presented in Table 4.

From the table it is evident that across all architectures and datasets *DTE* outperforms or performs as good as standard ensembling methods in terms of classification. This solidifies the argument that the combination of multiple expert models available to *DTE* conveys more information towards pattern recognition as compared to standard model ensembling procedures. The only exception here as well is with the *Skoda* dataset. This is because the standard ConvLSTM architecture in Skoda (without any forms of ensembling) was already outperforming *DTE*. Hence, the standard ConvLSTM ensemble also outperformed *DTE*. Interesting to note is that although it outperformed *DTE*, it did not improve the baseline classification metrics compared to the non-ensembled standard ConvLSTM model.

In terms of *ECE* an expected improvement is noticed in the standard ensemble models as compared to the baseline models. This is because the ensembles smoothens the overconfident softmax functions that is the output of a single neural network. When compared to *DTE* the **ECE** is quite similar.

### 4.6. Window-Size Selection

**Figure 11:**
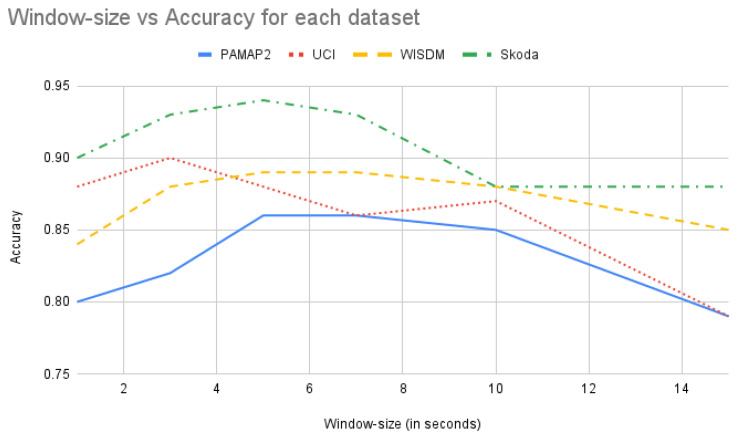
**Accuracy** (along *y*-axis) versus **window size** (in seconds along *x*-axis) using the best standard baseline architecture for all the datasets.

*DTE* needs a set of window sizes as input from which the temporal matrices are extracted for model training. In standard HAR the window size is usually chosen empirically for each dataset by experimenting with different values. The previous works provides us with the optimal window size for each of the datasets. In this work as well for each of the datasets, and a standard baseline neural network architecture, the accuracy of action recognition task is plotted with respect to different window sizes (in Figure 11).

This graph provides with an idea of the optimal window size for obtaining best performance for each workload and serves as a empirical foundation in selecting right window sizes for *DTE*. For construction of the *input ensembles* with different window sizes the following strategy is adopted: An equal number of values in both directions with uniform stepping from a chosen optimal window-size value. In the *PAMAP2* dataset the window size used in [30] is 5.12 s. From Figure 11 similar inferences can be drawn, i.e., the best accuracies are obtained around the 5 s window and 7 s window. It is also observed that for PAMAP2 dataset the accuracy starts improving from 3 s and it starts declining from 10 s, and after 14 s it drops below 0.8. Couple of window-size sets are created between 3 s till 12 s, and the classification matrices of *DTE* on those sets are tested, e.g., considering 5 s as an optimal time segment, and uniformly stepping on both sides of 5 s, a time-window set (of size 5) can be constructed with [3, 4, 5, 6, 7] s. In a similar manner, different window size sets are created with a different optimal time segment as mid point. These window-size sets are used for training different *DTE* models and for each window-size set the performance is observed. The results can be seen in Table 5.

It is seen that for *PAMAP2* the best performing window-size set is the one between 3 s to 7 s. Hence, in the final model this set is used for training. Similar experiments are conducted for the other datasets to obtain the best window sizes for temporal matrix extraction. From the experiments we observed that for *Skoda* and *UCI* datasets, the stepping from an optimal window size of 0.5 s is more suitable. Furthermore, for these two datasets lower window sizes are better (can be seen in Figure 11).

## 5. Future Work

While there are certain caveats and possible future works that could be associated with *DTE*, they can serve possible direction for future research. One central assumption of *DTE* is that the trained multiple models are strong learners. If a weak learner exists in the ensemble, it would negatively impact the overall classification and calibration performance. At this point, the window sizes for temporal sequence extraction are empirically chosen to ensure that the learners are strong. An interesting future direction to explore is an automatic adaptive selection of strong learners in the ensemble for *DTE*. Another downside of having an ensemble is the computational complexity that arises through training multiple models. Distillation of ensembles is a good direction to explore in this context [41]. These caveats expose some exciting research areas that could further improve the domain of reliable HAR. An interesting future direction would be to apply *DTE* on video-frame-based human activity recognition. The nature of the workload being time-series, *DTE* could be applied on video frames as well. The most popular architectures for video-based action recognition are spatio-temporal and 3D convolutions [42,43]. It could be promising to test the method on the architectural choices to deliver action recognition from videos. The present version of *DTE* is focused towards providing well-calibrated predictions. For which it uses a ensemble of models to deliver the inferences. In resource constrained environments where real-time inference is expected (e.g., live predictions using mobile or edge devices), the ensembles might overuse the resource and increase prediction latency. Thus a possible direction to explore is to provide calibrated response through lightweight modeling. Distillation of ensemble models [41] could be an interesting avenue to explore in this regards.

## 6. Conclusions

This work presents a novel way to incorporate the notion of confidence-calibrated predictions in human activity recognition with wearable sensors. The calibrated predictions representing the actual probability at outputs guarantees reliable modeling that can be safely incorporated into production pipelines, thus enabling safe, sustainable, and improved ubiquitous computing. While addressing the calibration problem, it is also made sure that the primary downstream task of HAR models, i.e., classification, is not hampered in any way.

The devised method called *deep time ensembles* can applied on any neural network architectures for HAR with sensor data. With a set of different window sizes, temporal matrices are extracted from raw sensor data. These temporal matrices are used to train individual models that are ensembled through an averaging procedure. Combining multiple expert models of the ensemble helps to calibrate the confidence of the softmax distribution and boost the classification measures.

To the best of our knowledge, no previous works have approached calibrating the predictive output of HAR. The approach is validated through extensive experiments on four benchmark datasets of activity recognition from different domains. Our calibration experiments show that in all the datasets, *deep time ensembles* outperform the calibration measures compared to the baseline models. Our classification experiments demonstrate that *DTE* boosts the classification performance for almost all the datasets. Thus, the promise of confidence calibrated reliable, as well as improved predictive performance, is delivered through *DTE*. To demonstrate the ability of the method in calibration, it is also compared with *temperature scaling*, a popular calibration method for deep learning [14]. Furthermore, our extensive experiments with a variety of DL architectures and datasets can also be used as a guideline for architecture selection in HAR. *DTE* will open a new research direction of calibrating HAR models as it demonstrates an easy way to obtain reliable confidence measures on wearable time-series input for HAR tasks.

## Figures and Tables

**Table 1:** Dataset, architectures, and macro f1-scores of the previous works forming our baselines.

Dataset	Previous Works	Architecture	Evaluation	F1-Score (Macro)
PAMAP2	Guan et al. [6]	LSTM	7 training/2 testing (and validation)	0.85
	Hammerla et al. [30]	CNN	7 training/2 testing (and validation)	0.83
WISDM	Ignatov et al. [20]	CNN	26 training/10 testing	0.90
	Agarwal et al. [29]	LSTM	0.7 training/0.3 testing	0.95
UCI	Ignatov et al. [20]	CNN	26 training/10 testing	0.93
Skoda	Adopted from Hammerla et al. [30]	CNN	0.8 training/0.2 testing	0.86
	LSTM-s model of Hammerla et al. [30]	LSTM	0.8 training/0.2 testing	0.84
	Ordonez et al. [21]	ConvLSTM	0.8 training/0.2 testing	0.92

**Table 2:** Number of temporal sequences and labels (for training) per dataset for each model in the ensemble.

Dataset	Time Window (In Seconds)	No. of Temporal Sequences (Train)	Number of Test Samples
PAMAP2	9	9658	83,031
	8	11,268	
	7	13,523	
	6	16,904	
	5	22,541	
UCI	3.5	4124	2993
	3	5184	
	2.5	6818	
	2	8677	
	1.5	11,774	
WISDM	10	7367	3026
	9	8230	
	8	9303	
	7	10,703	
	6	12,554	
Skoda	6	4780	23,157
	5.5	5039	
	5	5327	
	4.5	5484	
	4	5650	

**Table 3:** Classification and calibration results (10 experiments per setting) for baseline architectures versus *DTE* (our method) across all datasets.

Dataset	Architecture	F1${}_{m}$	F1${}_{w}$	Accuracy	ECE
PAMAP2	CNN (from Hammerla et al. [30])	0.79 ± 0.04	0.85 ± 0.03	0.86 ± 0.02	0.06 ± 0.01
	*DTE CNN*	0.83 ± 0.03	**0.89 ± 0.01**	**0.89 ± 0.01**	**0.03 ± 0.005**
	LSTM (from Guan et al. [6])	0.84 ± 0.02	0.85 ± 0.02	0.85 ± 0.02	0.08 ± 0.02
	*DTE LSTM*	**0.89 ± 0.01**	**0.89 ± 0.01**	**0.9 ± 0.009**	0.04 ± 0.008
UCI	CNN (from Ignatov et al. [20])	0.93 ± 0.004	0.93 ± 0.004	0.94 ± 0.004	0.04 ± 0.005
	*DTE CNN*	**0.94 ± 0.003**	**0.94 ± 0.003**	**0.95 ± 0.003**	**0.02 ± 0.004**
	LSTM	0.91 ± 0.02	0.92 ± 0.009	0.92 ± 0.008	0.04 ± 0.003
	*DTE LSTM*	**0.94 ± 0.003**	**0.94 ± 0.004**	0.94 ± 0.004	**0.02 ± 0.001**
WISDM	CNN (from Ignatov et al. [20])	0.87 ± 0.01	0.91 ± 0.01	0.91 ± 0.01	0.09 ± 0.007
	*DTE CNN*	0.88 ± 0.01	**0.93 ± 0.01**	0.92 ± 0.01	0.04 ± 0.008
	LSTM (from Agarwal et al. [29])	0.89 ± 0.01	0.9 ± 0.01	0.89 ± 0.01	0.05 ± 0.009
	*DTE LSTM*	**0.91 ± 0.008**	**0.93 ± 0.006**	**0.93 ± 0.007**	**0.03 ± 0.01**
Skoda	LSTM (LSTM-s model from [30])	0.83 ± 0.02	0.88 ± 0.01	0.89 ± 0.01	0.06 ± 0.003
	*DTE LSTM*	0.86 ± 0.004	0.89 ± 0.001	0.89 ± 0.001	**0.03 ± 0.002**
	CNN (from Hammerla et al. [30])	0.85 ± 0.01	0.88 ± 0.01	0.89 ± 0.01	0.06 ± 0.003
	*DTE CNN*	0.86 ± 0.002	0.89 ± 0.001	0.90 ± 0.001	0.04 ± 0.002
	ConvLSTM (from Ordonez et al. [21])	0.92 ± 0.02	**0.95 ± 0.01**	0.93 ± 0.01	0.08 ± 0.003
	*DTE ConvLSTM*	**0.93 ± 0.003**	0.94 ± 0.01	**0.94 ± 0.004**	**0.03 ± 0.003**

**Table 4:** Classification and calibration results (10 experiments per setting) for comparing standard ensemble versus *DTE* (our method) across all datasets and architectures.

Dataset	Architecture	F1${}_{m}$	F1${}_{w}$	Accuracy	ECE
PAMAP2	Ensemble CNN	0.78 ± 0.04	0.85 ± 0.04	0.86 ± 0.03	0.04 ± 0.002
	*DTE CNN*	0.83 ± 0.03	**0.89 ± 0.01**	0.89 ± 0.01	**0.03 ± 0.005**
	Ensemble LSTM	0.83 ± 0.01	**0.89 ± 0.02**	0.88 ± 0.04	0.04 ± 0.004
	*DTE LSTM*	**0.89 ± 0.01**	**0.89 ± 0.01**	**0.9 ± 0.009**	0.04 ± 0.008
UCI	Ensemble CNN	0.93 ± 0.03	0.93 ± 0.005	0.93 ± 0.004	0.03 ± 0.009
	*DTE CNN*	**0.94 ± 0.003**	**0.94 ± 0.003**	**0.95 ± 0.003**	**0.02 ± 0.004**
	Ensemble LSTM	0.93 ± 0.005	0.93 ± 0.007	0.93 ± 0.006	0.04 ± 0.009
	*DTE LSTM*	**0.94 ± 0.003**	**0.94 ± 0.004**	0.94 ± 0.004	**0.02 ± 0.001**
WISDM	Ensemble CNN	0.86 ± 0.01	0.9 ± 0.08	0.91 ± 0.06	0.04 ± 0.003
	*DTE CNN*	0.88 ± 0.01	**0.93 ± 0.01**	0.92 ± 0.01	0.04 ± 0.008
	Ensemble LSTM	0.9 ± 0.06	0.92 ± 0.03	0.92 ± 0.04	0.04 ± 0.002
	*DTE LSTM*	**0.91 ± 0.008**	**0.93 ± 0.006**	**0.93 ± 0.007**	**0.03 ± 0.01**
Skoda	Ensemble LSTM	0.84 ± 0.03	0.89 ± 0.01	0.89 ± 0.02	**0.03 ± 0.003**
	*DTE LSTM*	0.86 ± 0.004	0.89 ± 0.001	0.89 ± 0.001	**0.03 ± 0.002**
	Ensemble CNN	0.87 ± 0.03	0.91 ± 0.02	0.91 ± 0.03	0.04 ± 0.003
	*DTE CNN*	0.86 ± 0.002	0.89 ± 0.001	0.90 ± 0.001	0.04 ± 0.002
	Ensemble ConvLSTM	**0.93 ± 0.02**	**0.94 ± 0.01**	**0.95 ± 0.01**	0.04 ± 0.004
	*DTE ConvLSTM*	**0.93 ± 0.003**	**0.94 ± 0.01**	0.94 ± 0.004	**0.03 ± 0.003**

**Table 5:** HAR accuracy on different set of window sizes using *DTE* on the datasets using the best architectures (*DTE-LSTM* on PAMAP2, WISDM, *DTE-CNN* on UCI, and *DTE-ConvLSTM* on Skoda).

Dataset	Time-Window Sets (In Seconds)	Accuracy	F1 (Macro)	F1 (Average)
PAMAP2	[3, 4, 5, 6, 7]	0.9	0.86	0.88
	**[5, 6, 7, 8, 9]**	0.9	0.89	0.9
	[8, 9, 10, 11, 12]	0.87	0.82	0.85
UCI	**[1.5, 2, 2.5, 3, 3.5]**	0.94	0.94	0.95
	[2, 2.5, 3, 3.5, 4]	0.93	0.91	0.92
	[3.5, 4, 4.5, 5, 5.5]	0.89	0.87	0.9
WISDM	[3, 4, 5, 6, 7]	0.89	0.86	0.89
	**[6, 7, 8, 9, 10]**	0.92	0.9	0.92
	[8, 9, 10, 11, 12]	0.86	0.82	0.85
Skoda	[2, 2.5, 3, 3.5, 4]	0.91	0.86	0.9
	**[4, 4.5, 5,5. 5,6]**	0.93	0.93	0.94
	[6, 6.5, 7, 7.5, 8]	0.88	0.85	0.88

**Figure A4:**
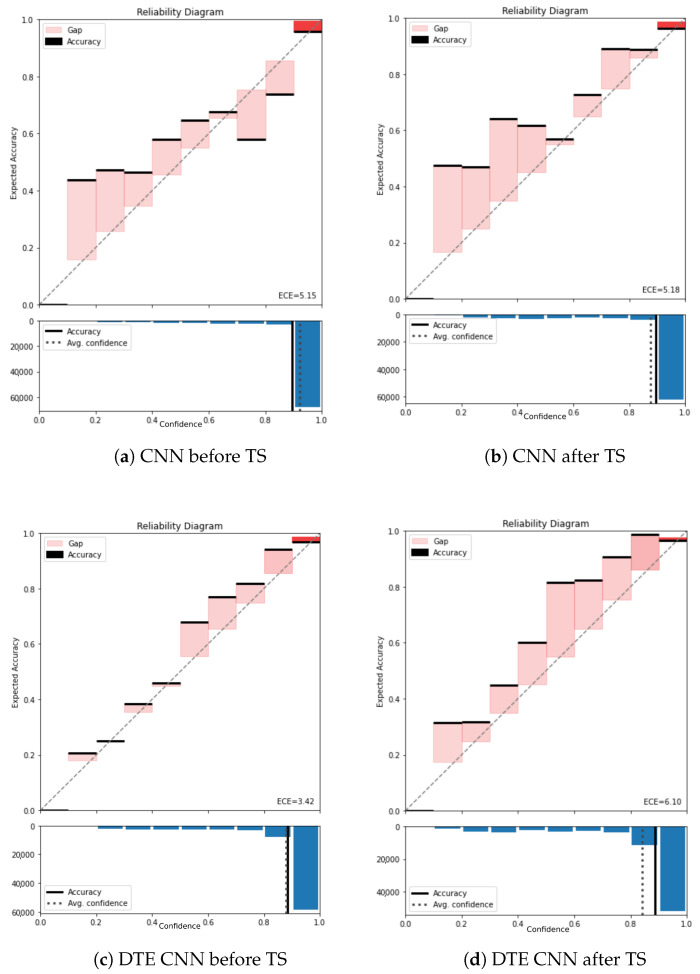
Reliability diagram of *PAMAP2* with and without temperature scaling for *CNN* and *DTE CNN*.

**Figure A5:**
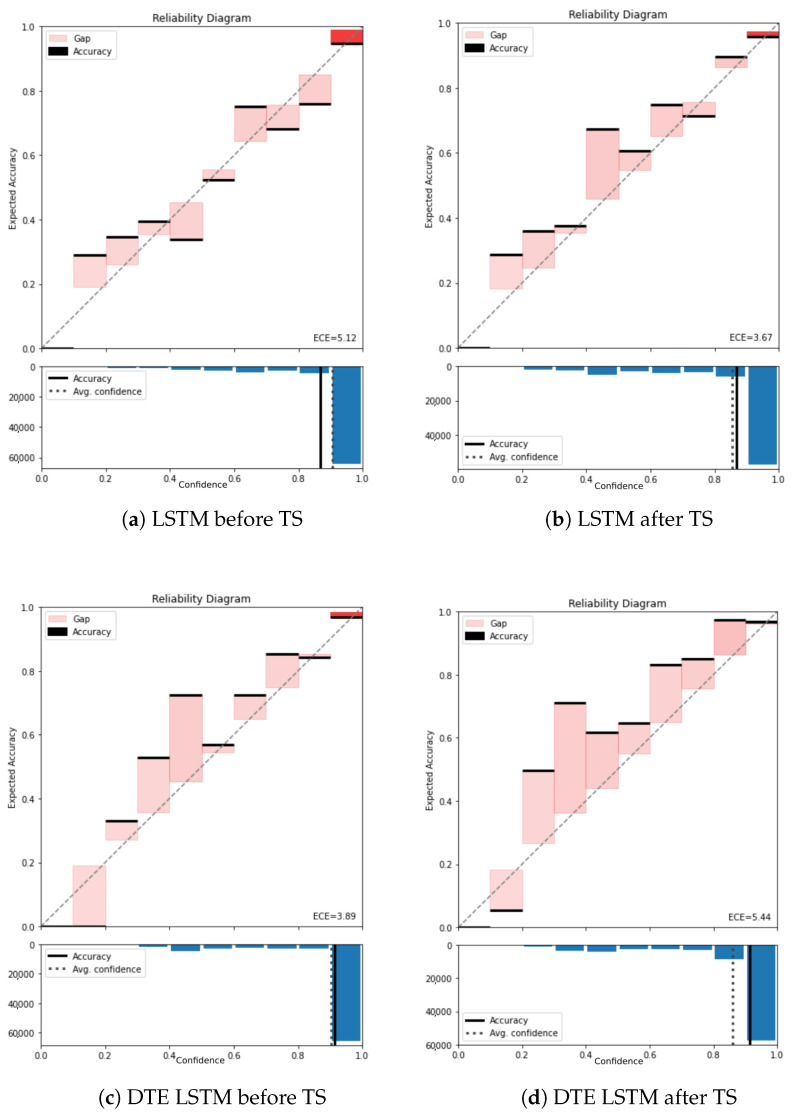
Reliability diagram of *PAMAP2* with and without temperature scaling for *LSTM* and *DTE LSTM*.

**Figure A6:**
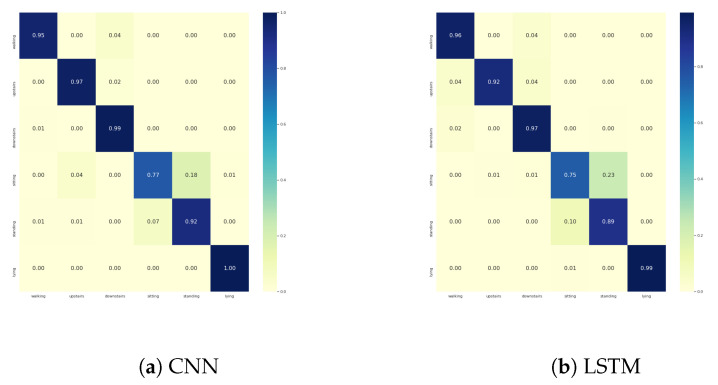
Confusion matrices of *UCI* with (**a**) CNN and (**b**) LSTM architectures.

**Figure A7:**
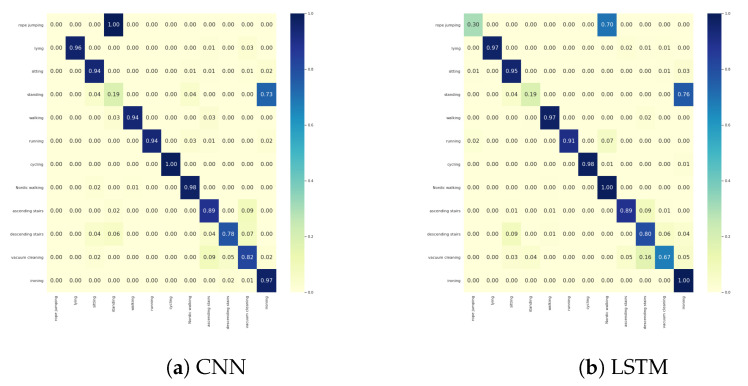
Confusion matrices of *PAMAP2* with (**a**) CNN and (**b**) LSTM architectures.

**Figure A8:**
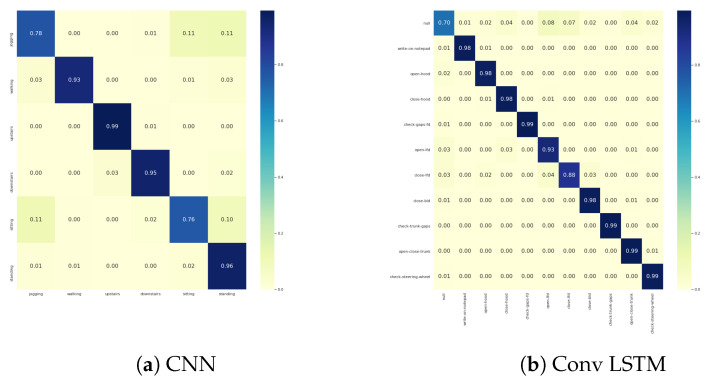
Confusion matrices of (**a**) WISDM CNN and (**b**) Skoda Conv LSTM classification.

**Figure A9:**
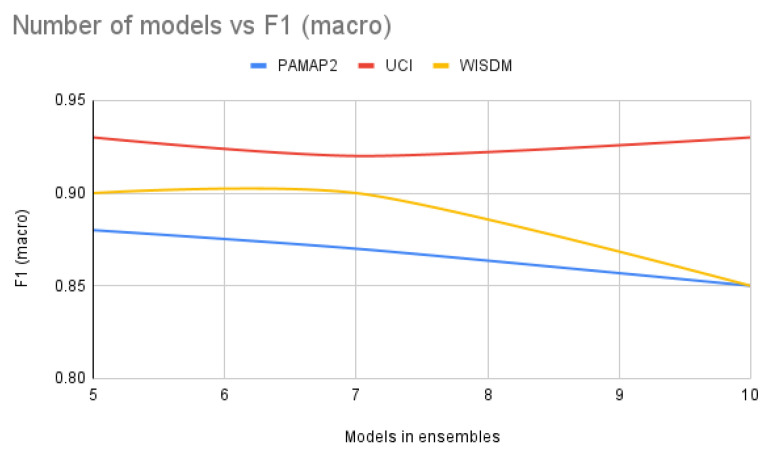
**Number of models** (along *x*-axis) versus **F1 (macro)** (along *y*-axis) using.

## Data Availability

Not applicable.

## Abbreviations

The following abbreviations are used in this manuscript:

AR	Activity Recognition
HAR	Human Activity Recognition
DTE	Deep Time Ensembles
CNN	Convolutional Neural Networks
LSTM	Long Short Term Memory
ECE	Expected Calibration Error

## Appendix A. Details of Implementation

The implementational details of each model are presented in this section. This is provided to assist in replicating our experiments and results.

**Table A1:** *PAMAP2 DTE-CNN* model configuration.

Category	Configuration
Window-sizes (s)	[5, 6, 7, 8, 9]
Batch size	64
Optimizer	Adam
Learning rate	0.00001
L2 regularization	0.005
Convolutional layer 1	filters: 128 filter size: 9 stride: 1 padding: valid activation: ReLU
Pooling layer	function: max size: 2 stride: 2
Dropout rate	0.1
Convolutional layer 2	filters: 128 filter size: 5 stride: 1 padding: valid activation: ReLU
Pooling layer	function: max size: 2 stride: 2
Dropout rate	0.25
Flatten and Dense layers	neurons: 512 activation: ReLU weight reg: L2
Dropout rate	0.5
Fully connected layer	neurons: number of classes activation: Softmax

**Table A2:** *PAMAP2 DTE-LSTM* model configuration.

Category	Configuration
Window-sizes (s)	[5, 6, 7, 8, 9]
Batch size	64
Optimizer	Adam
Learning rate	0.001
Dropout rate (before LSTM and Dense layers)	0.5
2 LSTM layers	neurons: 256
Fully connected layer	neurons: number of classes activation: Softmax

**Table A3:** *UCI DTE CNN* model configuration.

Category	Configuration
Window sizes (s)	[1.5, 2, 2.5, 3, 3.5]
Batch size	200
Optimizer	Adam
Learning rate	0.0005
L2 regularization	0.0005
Dropout rate	0.25
Convolutional layer	filters: 196 filter size: 16 stride: 1 padding: valid activation: ReLU bias init: 0.01 kernel init: TruncNorm(stddev = 0.01)
Pooling layer	function: max size: 4
Fully connected layer	neurons: 1024 activation: Softmax bias init: 0.01 weight init: TruncNorm(stddev = 0.01) kernel reg: L2

**Table A4:** *UCI DTE LSTM* model configuration.

Category	Configuration
Windows sizes	[1.5, 2, 2.5, 3, 3.5]
Batch size	200
Optimizer	Adam
Learning rate	0.001
LSTM layer 1	neurons: 128
LSTM layer 2	neurons: 128 return sequence: True
Fully connected layer	neurons: number of classes activation: Softmax

**Table A5:** *WISDM DTE CNN* model configuration.

Category	Configuration
Window sizes (s)	[6, 7, 8, 9, 10]
Batch size	200
Optimizer	Adam
Learning rate	0.0005
L2 regularization	0.0005
Dropout rate	0.25
Convolutional layer	filters: 196 filter size: 16 stride: 1 padding: valid activation: ReLU bias init: 0.01 kernel init: TruncNorm(stddev = 0.01)
Pooling layer	function: max size: 4
Fully connected layer	neurons: 1024 activation: Softmax bias init: 0.01 weight init: TruncNorm(stddev = 0.01) kernel reg: L2

**Table A6:** *WISDM DTE LSTM* model configuration.

Category	Configuration
Window sizes (s)	[6, 7, 8, 9, 10]
Batch size	64
Optimizer	Adam
Learning rate	0.0025
Learning loss rate	0.0015
2 LSTM layers	neurons: 30 bias init: 1
Fully connected layer	neurons: number of classes activation: Softmax

**Table A7:** *SKODA DTE ConvLSTM* model configuration.

Parameter	Configuration
Window sizes (s)	[4, 4.5, 5, 5.5, 6]
Batch size	100
Optimizer	RMSProp
Learning rate	0.001
Decay	0.9
Dropout rate (before LSTM and Dense layers)	0.5
3 Convolutional layers	filters: 64 filter size: 5 activation: ReLU
2 LSTM layers	SKODA DTE ConvLSTM model configurationneurons: 128
Fully connected layer	neurons: number of classes activation: Softmax

## Appendix B. Calibration Results

In this section we present the calibration results for datasets and architectures not presented in the main paper. The reliability diagrams demonstrate the calibration improvements by applying *DTE* to the remaining combinations of models and datasets.

### Appendix B.1. Calibration Results with Temperature Scaling

In this section, we show the reliability diagrams of *PAMAP2* dataset before and after temperature scaling for both CNN and LSTM architecture. As stated before, we initialized the hyperparameter T = 1.5 and tuned it using a validation set in order to smooth the softmax of the baseline models and improve their calibration. For the CNN architecture, the temperature scaling with T = 1.5 worsens the overall calibration (see Figure A4). We observe that in both cases (with and without *DTE*) the highest bins becomes better calibrated with temperature scaling. Thus, in this scenario TS calibrates the bins where most examples are concentrated at the cost of miscalibrating the lower bins. Furthermore since *DTE* assigns more examples to the lower bins due to the averaging process, the miscalibration of the lower bins become more pronounced in case of *DTE CNN* resulting in the highest **ECE** of 6.10.

The model that improved considerably after temperature scaling was the LSTM model trained using PAMAP2 dataset. As shown in Figure A5a,b ECE improved from 5.12% to 3.67%. The *DTE* is calibrated in itself in this case as well and exhibits more calibration error with temperature scaling.

We draw attention to the fact that the reliability diagrams for this section was adopted from one of the many experiments repeated towards robustness. Hence, the values of **ECE** might slightly differ from what we see in the Evaluation section of the paper.

### Appendix B.2. Classification Results

In this section we present the confusion matrices of all the classification results that we obtained through our experiments (except the ones already presented). For *UCI* dataset the confusion matrix is given in Figure A6.

For *PAMAP2* dataset the confusion matrix is given in Figure A7.

The confusion matrix for *WISDM* and *Skoda* dataset for *LSTM* and *Conv LSTM* architecture respectively is given in Figure A8.

## Appendix C. Number of Ensemble Models

In this section the graph in Figure A9 provides an intuition of the number of ensemble models that are required for *DTE*. As seen for most of the workloads increasing the size of the ensemble has almost no effect on the accuracy of the F1 score. Hence, for the current setting it is better to stick to a lesser number of models as it will lead to faster inference.
